# A cross-sectional study: Relationship between serum oxidative stress levels and arteriovenous fistula maturation in maintenance dialysis patients

**DOI:** 10.1515/med-2025-1149

**Published:** 2025-03-17

**Authors:** Mian Wang, JiHong Lian, Bin Kuang, ChaoHui Zhang, MingHui Zhang

**Affiliations:** Department of Vascular Surgery, Guangdong Provincial People’s Hospital (Guangdong Academy of Medical Sciences), Southern Medical University, Guangzhou, Guangdong, 510080, China; Department of Pathology, Guangdong Provincial People’s Hospital (Guangdong Academy of Medical Sciences), Southern Medical University, No.106, Zhongshan Second Road, Yuexiu District, Guangzhou, Guangdong, 510080, China

**Keywords:** maintenance dialysis, arteriovenous fistula maturation failure, oxidative stress, predictive efficacy

## Abstract

**Objective:**

This study was to investigate the relationship between serum superoxide dismutase (SOD) and malondialdehyde (MDA) and arteriovenous fistula (AVF) maturation in maintenance hemodialysis (MHD) patients.

**Methods:**

A total of 120 MHD patients were divided into a maturation group (72 patients) and a maturation failure group (48 patients). General information and ultrasound data were collected and venous blood was drawn for routine blood tests. General data and serum SOD and MDA were compared. Independent variables with statistically significant (*P* < 0.05) differences in univariate analysis were included in multivariate logistic regression. Serum SOD and MDA in predicting maturation failure of AVF were analyzed using receiver operating characteristic curves.

**Results:**

The internal diameter of the cephalic vein, internal diameter of the cephalic vein after tourniquet binding, internal diameter of the radial artery, and serum SOD level were lower and serum MDA level was higher in the maturation failure group. Reduced SOD and elevated MDA were independent risk factors for maturation failure of AVF. The area under the curve (AUC) for serum SOD and MDA was 0.68 each. When combined, their AUC for predicting AVF maturation failure was 0.79.

**Conclusion:**

Reduced serum SOD and increased MDA are risk factors affecting AVF maturation in MHD patients.

## 1 Introduction

Chronic kidney disease (CKD) is an irreversible and progressive kidney disease that is directly related to cardiovascular risk. Since 2017, the prevalence and mortality of CKD have been on the rise, resulting in significant social and medical burdens [1]. Maintenance hemodialysis (MHD) is an effective treatment for end-stage CKD. A well-functioning vascular access is a prerequisite to ensure the effect of dialysis, and arteriovenous fistula (AVF) can be the first choice of vascular access for MHD patients because of its longer use cycle, repeatable puncture, and lower infection rate [2]. Nevertheless, 20–60% of patients experience inadequate hemodialysis because of AVF maturation failure post-surgery, significantly impacting their prognosis [3]. Studies have been devoted to exploring the factors affecting AVF maturation, finding that arterial and venous vessel internal diameter, arterial blood flow, etc., are the main predictors of endovascular fistula maturation [4]. However, AVF maturation cannot yet be predicted by these factors. Therefore, further exploration of the factors affecting AVF maturation and early intervention are essential to improve AVF maturation rates and patient prognosis.

Oxidative stress is a major factor associated with AVF maturation failure, along with other causes [5]. Oxidative stress refers to a rise in oxidation levels within the body, characterized by an increase in reactive oxygen species (ROS) production. The ability to scavenge ROS, which is a part of anti-oxidation, cannot be substituted. Oxidative stress is prevalent in CKD, and hemodialysis is closely related to oxidative stress [6]. Peroxidation of membrane polyunsaturated fatty acids by free radicals produces molecules such as malondialdehyde (MDA), which can be used as an indicator to assess oxidative damage [7]. The pathological condition of CKD, along with patient characteristics and operation-related factors, affects the activity of oxidative stress factors and anti-oxidative stress factors in patients [8]. Sangeetha Lakshmi et al. showed a significant increase in MDA in end-stage renal disease (ESRD) patients undergoing dialysis [9]. Similarly, Loughrey et al. found that MDA levels were significantly elevated in patients undergoing hemodialysis, while plasma concentrations of antioxidant markers were decreased [10]. In dialysis, the process is impacted by the inadequate removal of inflammatory transmitters, the biocompatibility of the system, and the depletion of antioxidants from the dialysate solution [11]. The production of a large number of MDA highly active molecules and reduction of superoxide dismutase (SOD) result in oxidation exceeding the antioxidant capacity, causing oxidative stress and damaging tissue cells. SOD is a member of the antioxidant enzyme family of biological systems and is the only enzyme that mediates superoxide anion, playing a crucial role in the collective oxidative and antioxidant balance. Its primary function is to protect against mitochondrial ROS damage [12]. SOD is less active in patients undergoing hemodialysis treatment [13]. Both SOD and MDA are recognized markers associated with oxidative stress and play roles in vascular remodeling and renal diseases. However, it is not known whether there is a correlation between serum oxidative stress levels and AVF maturation failure in patients undergoing MHD.

In this study, MDA and SOD related to oxidative stress were analyzed before and after AVF surgery in MHD patients.

## 2 Materials and methods

### 2.1 General information

MHD patients who underwent AVF surgery for the first time in Guangdong Provincial People’s Hospital from July 2019 to October 2023 were selected to participate in this study. The sample size was calculated using PASS11.0 multi-group comparison. Setting the test level *α* = 0.01, the test efficacy (1 − *β*) as 95%, and the failure rate as 10%, the sample sizes of the two groups were calculated to be at least 40 cases each. In order to improve the accuracy of the results of the study, and taking into account the actual situation of the hospital outpatient clinic and the time limit of the study, the sample size of the two groups was finally determined to be 120 cases. According to the patients’ postoperative AVF maturation they were divided into the maturation group and maturation failure group.

Inclusion criteria included: (1) patients had K/DOQI stage V CKD and underwent long-term hemodialysis (at least 3 months). (2) Patients were 18–60 years old. (3) The same surgical team performed the radial artery-cephalic vein anastomosis (RCF), known as arteriovenous fistuloplasty. A deep vein catheter was usually used as vascular access during the first 3 months. (4) The pre-surgery color Doppler ultrasound of the radial artery and cephalic vein satisfied the criteria for AVF: the depth of the vessel <6 mm, the internal diameter of the cephalic vein was ≥1.5 mm, and the internal diameter of the cephalic vein after tourniquet binding was >2 mm. (5) According to the ultrasound examination, the arteries and veins were open throughout the lumen, without any stenosis or thrombosis. (6) Patients gave informed consent and were willing to participate in the present study.

Exclusion criteria included: (1) patients with combined organic lesions and malignant tumors; (2) patients in the acute exacerbation of severe infections, fever, and chronic diseases; (3) patients with severe infectious diseases, systemic immune diseases, and neurological disorders; (4) patients reconstructed for AVF; and (5) patients whose data were incomplete. The study was approved by the Ethics Committee of Guangdong Provincial People’s Hospital, and all patients signed a written informed consent form.

### 2.2 Clinical information

General information was collected, including gender, age, and underlying diseases. All surgeries were performed by the same surgeon, who performed a preoperative evaluation of the proposed surgical site in all enrolled patients.

Ultrasonography (1–3 days before surgery): The examination was performed with HD7 color Doppler ultrasound machine with a frequency of 10 MHz. The inner diameter of the cephalic vein after tourniquet binding (the vein was blocked and filled with a tourniquet at 2 cm below the elbow, and the inner diameter of the cephalic vein was measured), the inner diameter of the radial artery, and the inner diameter of the cephalic vein were examined. When measuring the internal diameter of the cephalic vein and radial artery, the acoustic beam was aligned perpendicularly to the long axis of the vessels, and the average value was calculated from two measurements.

### 2.3 Laboratory indicators

Laboratory indicators were collected from all patients during the preoperative week. The patients were fasted for 10 h. Venous blood (4 mL) was collected using a vacuum blood tube and centrifuged at 3,000 rpm for 10 min, and the serum was separated and stored at −80°C. Lipid metabolism indices including high-density lipoprotein cholesterol (HDL-C), low-density lipoprotein cholesterol (LDL-C), total cholesterol (TC), and triacylglycerol (TG) were measured by using a Hitachi 7060 automated biochemical analyzer. SOD levels were measured by colorimetric assay. MDA levels were assessed using an ELISA kit (Beyotime, China) and optical density was read on a microplate reader (Thermo, USA) at 450 nm.

### 2.4 Arteriovenous fistuloplasty

Conventional disinfection and local infiltration anesthesia were performed. An incision was made between the radial artery and the cephalic vein. The blood flow of the radial artery and the cephalic vein was blocked. The distal end of the cephalic vein was ligated, and the lateral wall of the radial artery was cut longitudinally for 6–8 mm and rinsed with heparin saline. The radial artery and the cephalic vein were continuously sutured with a 7-0 polypropylene non-absorbable thread. No significant bleeding occurred, and the blood flow was adequate. The skin was sutured layer by layer and covered with gauze.

### 2.5 AVF maturation determination

AVF maturation is determined by the relevant criteria in the “Chinese expert consensus on vascular access for hemodialysis” [14]: (1) The tremor at the anastomotic orifice is stable, with no abnormal enhancement, weakening, or disappearance. Veins in the fistula segment are straight and superficial, allowing for easy puncture, with uniform thickness and sufficient puncture area, and the wall of the fistula vessel is well elastic, and the tremor can be palpated without enhancement, weakening, or disappearance. (2) Dialysis pump-controlled blood flow of 200 mL/min or more.

Doppler ultrasonography was used for those who did not undergo dialysis within 8 weeks. The diameter of the cephalic vein ≥5 mm, blood flow ≥500 mL/min, and the depth from the skin <6 mm met the criteria for AVF maturation.

### 2.6 Data analysis

All data were statistically analyzed using SPSS 26.0 software. Normality test was performed by Shapiro–Wilk method. Information on continuous variables that conformed to normal distribution was described by mean + standard deviation (SD), and comparisons between groups were made using Student’s *t*-test. Qualitative information was expressed as the number of cases and percentage, and the chi-square test was used. Multifactorial logistic regression was used to analyze the factors influencing AVF maturation failure in patients. The value of SOD and MDA levels in predicting AVF maturation failure in patients was assessed using the receiver operating characteristic (ROC) curve and area under the curve (AUC). MedCalc software was used to compare whether there was a statistically significant difference in AUC between the different indicators. *P* < 0.05 was considered statistically significant.


**Informed consent:** Written informed consent was provided by all patients prior to the study start.
**Ethical approval:** The present study was approved by the Ethics Committee of Guangdong Provincial People’s Hospital (No. 201801GD-25) All procedures were performed in accordance with the ethical standards of the Institutional Review Board and the Declaration of Helsinki, and its later amendments or comparable ethical standards.

## 3 Results

### 3.1 Comparison of clinical general information between the maturation group and maturation failure group

There were 72 cases in the maturation group, of which 39 were males and 33 were females; their ages ranged from 39 to 54 years, with a mean age 47.11 ± 7.52 years. There were 48 cases in the maturation failure group, including 22 males and 26 females. The age ranged from 40 to 58 years, with an average age of 49.52 ± 8.56 years. The internal diameter of the cephalic vein, internal diameter of the cephalic vein after tourniquet binding, and internal diameter of the radial artery in the maturation failure group were smaller than those in the maturation group (*P* < 0.05). Gender, age, comorbidity, lipid metabolism indices, fasting blood glucose, blood calcium, blood phosphorus, and blood creatinine in the two groups were not statistically significant (*P* > 0.05, Table 1).

**Table 1:** Comparison of clinical general data between maturation group and maturation failure group

Characteristics	Maturation group (*n* = 72)	Maturation failure group (*n* = 48)	*P*
Male no.	34 (47%)	22 (46%)	0.854
Age (year)	47.11 ± 7.52	49.52 ± 8.56	0.11
**Comorbidity**			
Specific type of kidney disease	33 (46%)	19 (40%)	0.57
Diabetic nephropathy	22 (30%)	13 (27%)	0.84
Hypertensive renal disease	25 (35%)	10 (21%)	0.15
Calcium (mmol/L)	1.91 ± 0.41	1.97 ± 0.59	0.51
Serum phosphate (mmol/L)	1.65 ± 0.58	1.71 ± 0.34	0.52
Creatinine (μmol/L)	649.52 ± 24.13	657.05 ± 20.41	0.08
Bloodglucose (mmol/L)	6.07 ± 1.12	6.30 ± 1.02	0.26
TC (mmol/L)	2.05 ± 0.26	2.14 ± 0.44	0.16
Triglycerides (mmol/L)	1.55 ± 0.57	1.62 ± 0.52	0.50
HDL-C (mmol/L)	1.12 ± 0.21	1.06 ± 0.26	0.17
LDL-C (mmol/L)	1.90 ± 0.45	1.74 ± 0.55	0.08
Cephalic vein diameter (mm)	2.85 ± 0.31	2.63 ± 0.21	<0.01
Cephalic vein diameter behind bundle arm (mm)	3.32 ± 0.33	3.17 ± 0.34	0.02
Radial artery diameter (mm)	2.52 ± 0.42	2.35 ± 0.26	0.01

Data are expressed as mean ± SD or *N* (%). Continuous variables that meet the normal test were tested by student’s *t*-test, and categorical variables were tested by chi-square test.

### 3.2 Comparison of serum SOD and MDA levels between the maturation group and maturation failure group

Serum MDA levels were 1.08-fold higher (*P* < 0.05) in the maturation failure group than in the maturation group. Serum SOD levels were lower in the maturation failure group than in the maturation group (*P* < 0.05, Table 2). Serum SOD levels were 1.06 times higher in the maturation group compared to the maturation failure group.

**Table 2:** Comparison of serum SOD and MDA levels in two groups of patients

Groups	*N*	SOD (nmol/mL)	MDA (nmol/mL)
Maturation group	72	5.60 ± 0.65	5.68 ± 0.66
Maturation failure group	48	5.29 ± 0.29	6.14 ± 0.70
*P* value	**–**	0.00	<0.01

Data were expressed as mean ± SD. Student’s *t*-test was used for statistical analysis. Abbreviations: SOD, superoxide dismutase; MDA, malondialdehyde.

### 3.3 Multifactorial logistic regression analysis of independent influences on AVF maturation failure in MHD patients

**Figure 1:**
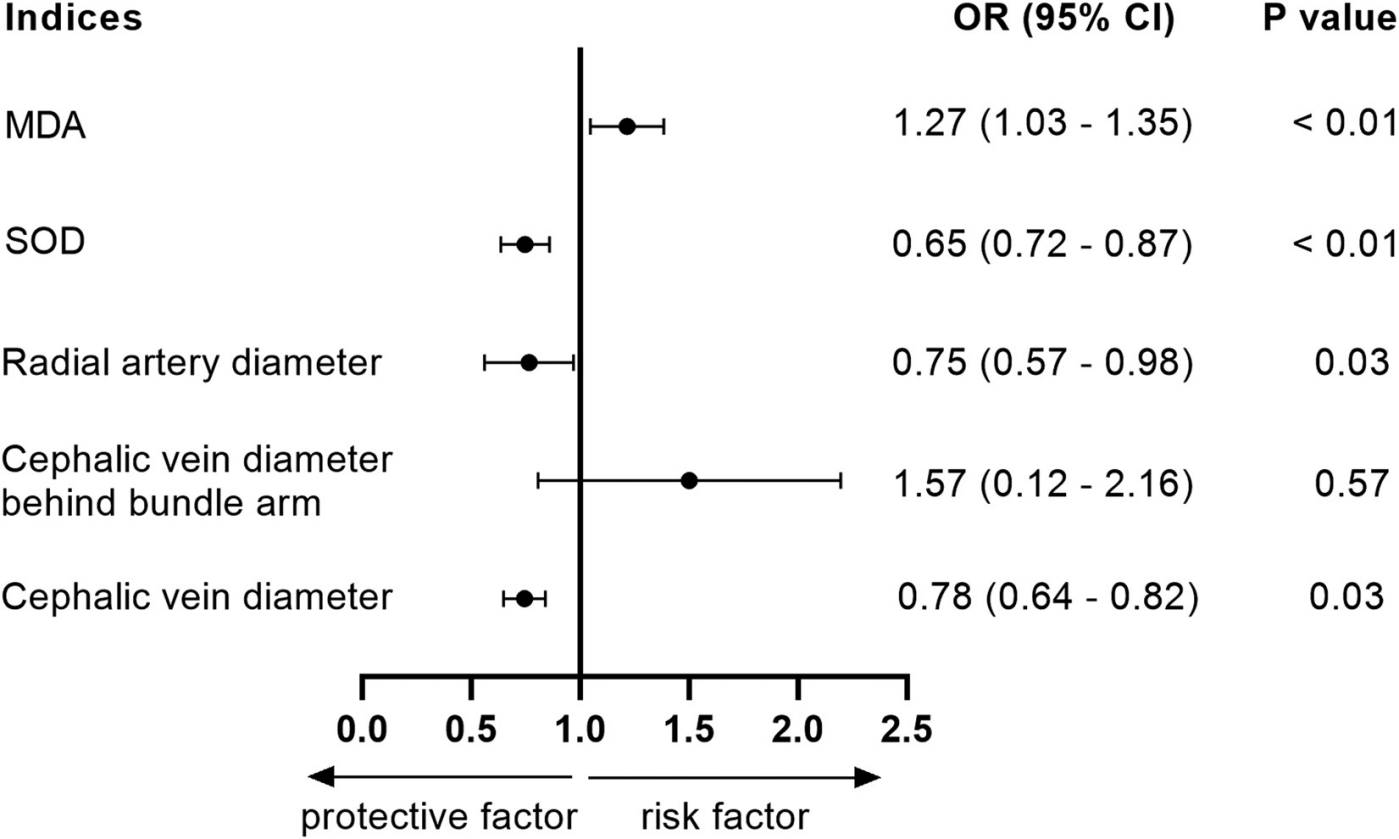
A tree diagram based on multifactorial logistic regression analysis. Elevated serum SOD and MDA levels are independent risk factors for AVF maturation failure.

A multifactorial logistic regression model was established with the factors analyzed in Tables 1 and 2 as independent variables (*P* < 0.05), whereas diabetic nephropathy, internal diameter of the cephalic vein, internal diameter of the cephalic vein after tourniquet binding, internal diameter of the radial artery, serum levels of SOD and MDA, and maturation failure of AVF (categorical variables, yes = 1, no = 0) as dependent variables. The internal diameter of the cephalic vein after tourniquet binding (OR = 1.57, *P* = 0.57) was not an independent risk factor for AVF maturation failure. However, the levels of serum SOD (OR = 0.72, *P* < 0.01), the internal diameter of the cephalic vein (OR = 0.78, *P* = 0.03), and the increased internal diameter of the radial artery (OR = 0.75, *P* = 0.03) were independent protective factors, and serum MDA (OR = 1.27, *P* < 0.01) was an independent risk factor influencing AVF maturation failure *(P* < 0.05, Figure 1).

### 3.4 ROC curve assessment of the predictive efficacy of serum SOD and MDA levels for AVF maturation failure in MHD patients

**Figure 2:**
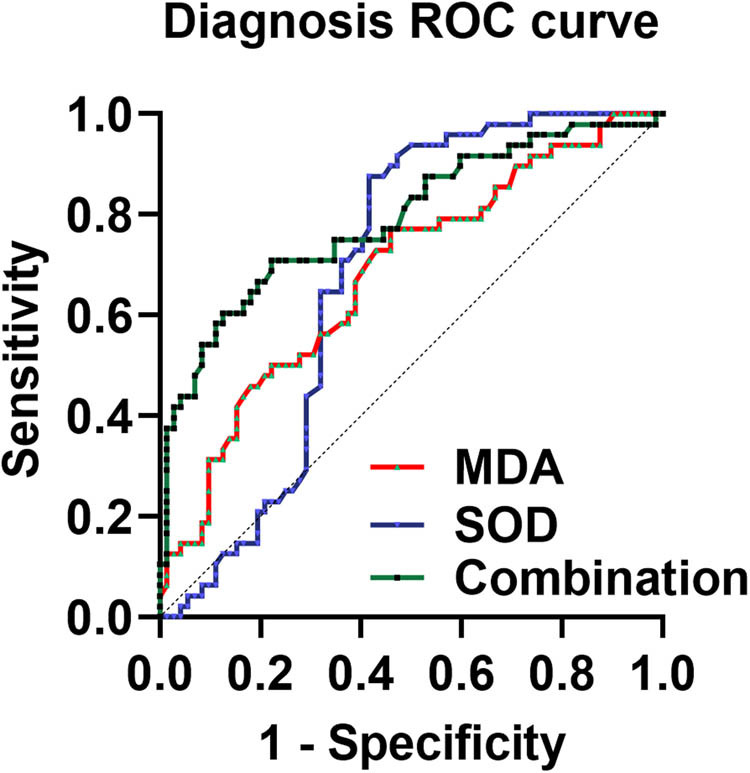
ROC curve results shows that serum SOD and MDA levels were good predictors of AVF maturation failure in MHD patients.

The ROC curve was plotted with the maturation failure group as positive samples, the maturation group as negative samples, and AVF maturation failure as a state variable (1 = occurring, 0 = not occurring). The AUC of serum SOD was 0.68 (95% CI = 0.59–0.78, *P* < 0.05), and the sensitivity of predicting AVF maturation failure was 87.50% and the specificity was 58.33% when the cut-off value was taken to be SOD < 5.57 pg/mL. The AUC of MDA was 0.68 (95% CI = 0.58–0.78, *P* < 0.05). When the cut-off value was taken as MDA > 5.69 pg/mL, the sensitivity of predicting AVF maturation failure was 75.00% and the specificity was 54.17%. The AUC of combined SOD and MDA for predicting AVF maturation failure was higher than that of the single index, which was 0.79 (95% CI = 0.71–0.88, *P* < 0.05), with a sensitivity and specificity of 70.83 and 77.78%, respectively (Table 3, Figure 2).

**Table 3:** Predictive efficacy of serum SOD and MDA levels for AVF maturation failure in MHD patients as assessed by ROC curves

Indices	Sensitivity (%)	Specificity (%)	AUC	95% CI	*P*
SOD	87.50	58.33	0.68	0.59–0.78	0.00
MDA	77.08	54.17	0.68	0.58–0.78	0.00
Combination	70.83	77.78	0.79	0.71–0.88	<0.01

Abbreviations: SOD, superoxide dismutase; MDA, malondialdehyde; AVF, arteriovenous fistula; AUC, area under the curve; CI, confidence interval.

## 4 Discussion

Vascular access is a prerequisite for hemodialysis treatment. AVF is surgically anastomosed between the blood supply vessel and the draining vein. AVF matures through vasodilation, inhibition of neointima hyperplasia, outward remodeling, and dilation of the vascular lumen [15]. Gender, age, primary morbidity, and surgical technique are some of the factors affecting AVF maturation, but they cannot well-predict endovascular fistula maturation. Therefore, understanding the relevant factors affecting AVF maturation in patients with MHD and developing relevant interventions are essential to improve patient prognosis. This study showed that elevated serum levels of MDA and reduced levels of SOD were independent risk factors for AVF maturation failure in patients, and the efficacy of the two metrics in combination in predicting AVF maturation was better.

AVF maturation involves outward remodeling of the vessel and thickening of the vessel wall. When AVF is established, outflow veins are exposed to the high flow, high shear stress, high pressure, and oxygen-rich environment of the artery. The high flow and high shear stress of inflow arteries and outflow veins adapting to the arterial environment can cause outward remodeling of the vessel (especially venous expansion). Adaptation of inflow arteries and outflow veins to the arterial environment of high pressure, high shear stress, and tensile stress can lead to thickening of the vessel wall [16,17]. Oxidative stress is prevalent in CKD patients and progressively worsens with the years of hemodialysis in uremic patients [18]. Oxidative stress is also associated with diabetes, inflammation, and progression of CKD to ESRD, and is an independent predictor of death and development of complications in patients with MHD [19,20]. Oxidative stress is exacerbated in advanced stages of CKD and is more severe in patients with ESRD on MHD therapy, and likewise has an impact on the maturation of AVF.

Diabetes and CKD share a common underlying pathogenic process that leads to the accumulation of free radical products. Dursun et al. studied the effects of hemodialysis and diabetes on oxidative stress and found that both diabetes and end-stage CKD induced oxidative activity [21]. Lipid peroxidation and glucose oxidation are found to be closely associated with accelerated coronary vascular calcification in MHD patients. Elevated TG levels are also associated with oxidative stress in CKD [22–24]. In addition, intravascular diameter has been reported to be a major factor influencing AVF maturation. Small radial artery diameters result in poor postoperative blood flow, which fails to meet the blood flow criteria required for endovascular fistula maturation, subsequently increasing the incidence of poor endovascular fistula maturation [25–27]. Parmar et al. concluded that the risk of AVF dysfunction was 50% when the radial artery diameter was less than 1.5 mm [28]. In the present study, diabetes mellitus and CKD as comorbidities as well as glycemic and lipid-related factors were included to explore the factors influencing AVF maturation failure. In this study, we first compared the differences between the maturation group and the maturation failure group in terms of various clinical data, and it was clear that patients with AVF maturation failure developed vascular stenosis.

MDA is a predictor of survival in patients with MHD and has been shown to be significantly elevated in the serum of patients with MHD. Increases in MDA lead to increased morbidity and mortality in patients with MHD, a process that may begin in the early stages of renal failure through ischemic glomerular and tubular injury, malnutrition, loss of antioxidants during dialysis, exacerbation of bacterial or toxic products in the dialysate, and hypersensitivity reactions [18,20,29]. People suffering from MHD often find themselves in an inflammatory environment, where large molecular toxins and oxidative stressors such as MDA accumulate in the body over time and are hard to remove effectively [30,31]. SOD protects cells from damage by scavenging oxygen radicals, which plays a crucial role in the oxidative and antioxidant balance of the body. When the level of oxidative stress is increased in the body, MDA is significantly enhanced and SOD is decreased. This is consistent with the findings of the present study that there is a correlation between SOD and MDA levels and AVF maturation, and that the level of oxidative stress increases significantly with the aggravation of the disease, and MDA and SOD change accordingly. Multifactorial logistic regression analysis revealed that SOD and MDA were independent risk factors for AVF maturation failure, suggesting that SOD and MDA may be potential biomarkers for predicting AVF maturation. Altered chromogenic amino acid metabolism, tyrosine and phenylalanine metabolism, and arginine metabolism can be seen in patients with CKD stage 5 [32,33]. Tryptophan metabolism produces indolephenol sulfate and allantoic acid, the accumulation of which impairs endothelial cell function and promotes oxidative stress [34–36]. Elevated levels of oxidative stress result in a high level of mono-oxygenation. Elevated levels of oxidative stress lead to the breakdown of nitric oxide (NO). When NO decreases, the vascular endothelium loses its ability to protect the vessel wall, leading to abnormal proliferation and migration of vascular smooth muscle cells, resulting in abnormal vascular remodeling, exacerbating intimal hyperplasia, causing vascular stenosis and thrombosis, and impairing AVF maturation [37,38]. In clinical practice, monitoring serum SOD and MDA levels can help assess oxidative stress status in MHD patients and may be used to predict AVF maturation. By identifying those patients who may be at risk for poor AVF maturation, physicians can take appropriate preventive measures, such as adjusting anti-inflammatory treatments, optimizing dialysis parameters, or implementing other vasoprotective strategies, to improve the success and durability of AVF. In addition, understanding the role of oxidative stress in AVF maturation could also help in the development of new therapeutic approaches to improve vascular access in patients with MHD.

This study is a single-center study with a small sample size. In the future, we will conduct a multicenter study with larger sample size to search for new oxidative stress-related biomarkers using metabolomics, proteomics, and epigenetics perspectives; further analyze in depth the relationship between SOD, MDA, and other oxidative stress-related factors and AVF maturation failure in patients with MHD and their prognosis. There may be some potential confounders in this study, such as patients’ age, gender, lifestyle, etc., whose presence may distort the true relationship between the index and the disease. In this regard, in future studies we will randomize the sample as much as possible so that the confounders are evenly distributed among the groups. Second, we will collect as much information as possible about possible confounders. In-depth stratified and multivariate analyses to include confounders as covariates in regression models are also needed to minimize data bias in the future. As an observational study, this study can only preliminarily analyze the correlation between SOD and MDA and AVF maturation failure, and cannot clarify the causal relationship and possible mechanisms, which need to be confirmed by further follow-up studies.

The number of CKD patients beginning hemodialysis is rising, and the dialysis demographic is progressively younger. This research suggests that serum SOD and MDA could serve as novel biomarkers for predicting AVF maturation failure, offering a fresh perspective on AVF maturation and maintenance.
